# Plasma proteomic signatures of a direct measure of insulin sensitivity in two population cohorts

**DOI:** 10.1007/s00125-023-05946-z

**Published:** 2023-06-17

**Authors:** Daniela Zanetti, Laurel Stell, Stefan Gustafsson, Fahim Abbasi, Philip S. Tsao, Joshua W. Knowles, RISC Investigators, Ele Ferrannini, Michaela Kozakova, Amalia Gastaldelli, Simon Coppack, Beverley Balkau, Jacqueline Dekker, Mark Walker, Andrea Mari, Andrea Tura, Martine Laville, Henning Beck, John Nolan, Geremia Bolli, Alain Golay, Thomas Konrad, Peter Nilsson, Olle Melander, Geltrude Mingrone, Colin Perry, John Petrie, Michael Krebs, Rafael Gabriel, Asimina Mitrakou, Piermarco Piatti, Nebojsa Lalic, Marku Laakso, Björn Zethelius, Johan Ärnlöv, Beverley Balkau, Mark Walker, Laura C. Lazzeroni, Lars Lind, John R. Petrie, Themistocles L. Assimes

**Affiliations:** 1 Department of Medicine, Division of Cardiovascular Medicine, Stanford University School of Medicine, Stanford, CA USA; 2 VA Palo Alto Health Care System, Palo Alto, CA USA; 3 Department of Biomedical Data Science, Stanford University School of Medicine, Stanford, CA USA; 4 Department of Medical Sciences, Uppsala University, Uppsala, Sweden; 5 Stanford Diabetes Research Center, Stanford University School of Medicine, Stanford, CA USA; 6 Stanford Cardiovascular Institute, Stanford University School of Medicine, Stanford, CA USA; 7 Stanford Prevention Research Center, Stanford University School of Medicine, Stanford, CA USA; 8 Department of Public Health/Geriatrics, Uppsala University, Uppsala, Sweden; 9 Division of Family Medicine and Primary Care, Department of Neurobiology, Care Sciences and Society, Karolinska Institute, Stockholm, Sweden; 10 Department of Health and Social Studies, Dalarna University, Falun, Sweden; 11 Clinical Epidemiology, Centre for Research in Epidemiology and Population Health, Inserm U1018, Villejuif, France; 12 Translational and Clinical Research Institute, Newcastle University, Newcastle upon Tyne, UK; 13 Department of Psychiatry and Behavioral Sciences, Stanford University, Stanford, CA USA; 14 School of Health and Wellbeing, College of Medical, Veterinary and Life Sciences, University of Glasgow, Glasgow, UK; 15 Department of Epidemiology and Population Health, Stanford University School of Medicine, Stanford, CA USA

**Keywords:** Euglycaemic–hyperinsulinaemic clamp, Insulin resistance, Insulin sensitivity, LASSO, Plasma proteomics, Population study, Stability selection

## Abstract

**Aims/hypothesis:**

The euglycaemic–hyperinsulinaemic clamp (EIC) is the reference standard for the measurement of whole-body insulin sensitivity but is laborious and expensive to perform. We aimed to assess the incremental value of high-throughput plasma proteomic profiling in developing signatures correlating with the *M* value derived from the EIC.

**Methods:**

We measured 828 proteins in the fasting plasma of 966 participants from the Relationship between Insulin Sensitivity and Cardiovascular disease (RISC) study and 745 participants from the Uppsala Longitudinal Study of Adult Men (ULSAM) using a high-throughput proximity extension assay. We used the least absolute shrinkage and selection operator (LASSO) approach using clinical variables and protein measures as features. Models were tested within and across cohorts. Our primary model performance metric was the proportion of the *M* value variance explained (*R*^2^).

**Results:**

A standard LASSO model incorporating 53 proteins in addition to routinely available clinical variables increased the *M* value *R*^2^ from 0.237 (95% CI 0.178, 0.303) to 0.456 (0.372, 0.536) in RISC. A similar pattern was observed in ULSAM, in which the *M* value *R*^2^ increased from 0.443 (0.360, 0.530) to 0.632 (0.569, 0.698) with the addition of 61 proteins. Models trained in one cohort and tested in the other also demonstrated significant improvements in *R*^2^ despite differences in baseline cohort characteristics and clamp methodology (RISC to ULSAM: 0.491 [0.433, 0.539] for 51 proteins; ULSAM to RISC: 0.369 [0.331, 0.416] for 67 proteins). A randomised LASSO and stability selection algorithm selected only two proteins per cohort (three unique proteins), which improved *R*^2^ but to a lesser degree than in standard LASSO models: 0.352 (0.266, 0.439) in RISC and 0.495 (0.404, 0.585) in ULSAM. Reductions in improvements of *R*^2^ with randomised LASSO and stability selection were less marked in cross-cohort analyses (RISC to ULSAM *R*^2^ 0.444 [0.391, 0.497]; ULSAM to RISC *R*^2^ 0.348 [0.300, 0.396]). Models of proteins alone were as effective as models that included both clinical variables and proteins using either standard or randomised LASSO. The single most consistently selected protein across all analyses and models was IGF-binding protein 2.

**Conclusions/interpretation:**

A plasma proteomic signature identified using a standard LASSO approach improves the cross-sectional estimation of the *M* value over routine clinical variables. However, a small subset of these proteins identified using a stability selection algorithm affords much of this improvement, especially when considering cross-cohort analyses. Our approach provides opportunities to improve the identification of insulin-resistant individuals at risk of insulin resistance-related adverse health consequences.

**Graphical Abstract:**

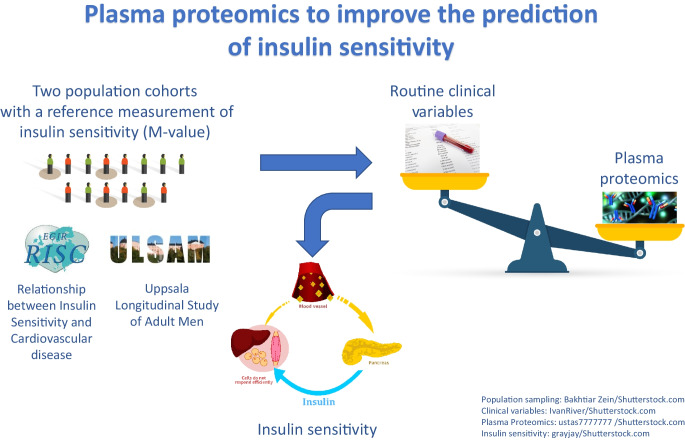

**Supplementary Information:**

The online version contains peer-reviewed but unedited supplementary material available at 10.1007/s00125-023-05946-z.


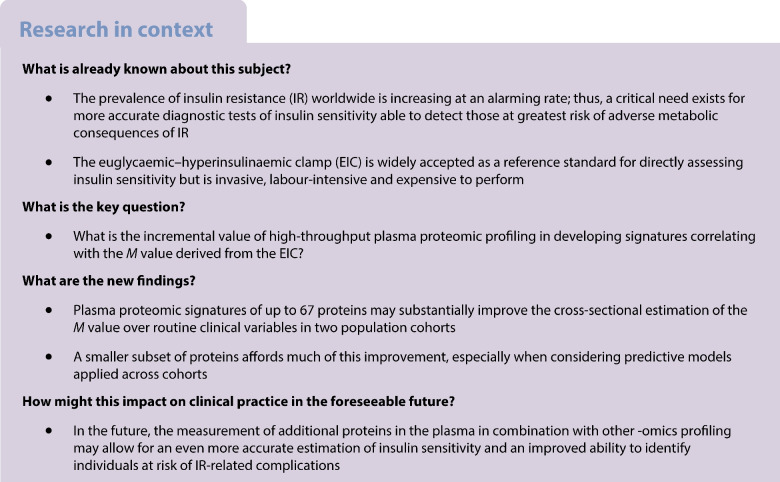


## Introduction

Insulin action has been quantitatively defined as its ability to regulate glucose disposal [1]. Insulin resistance (IR) is a physiological state whereby glucose disposal is impaired and accompanied by a compensatory hyperinsulinaemia [1]. IR is a primary risk factor for the development of type 2 diabetes and non-alcoholic fatty liver disease [1]. Through the promotion of atherogenic dyslipidaemia and hypertension, IR is a strong risk factor for atherosclerotic CVD (ASCVD) [1].

The euglycaemic–hyperinsulinaemic clamp (EIC) is widely accepted as the reference standard for directly assessing insulin sensitivity [2, 3]. During an EIC, the plasma insulin concentration is acutely raised and maintained at a constant level by a primed continuous infusion of insulin [2, 3]. The plasma glucose concentration is held constant at basal levels by a variable rate glucose infusion using the negative feedback principle [2, 3]. Under these steady-state conditions of euglycaemia, the glucose infusion rate (‘*M* value’) equals glucose uptake by all the tissues in the body and is therefore a measure of tissue sensitivity to exogenous insulin [2, 3]. Insulin sensitivity as estimated by the EIC has been linked to incident type 2 diabetes [4], ASCVD [5, 6] and heart failure [7], but is invasive, labour-intensive and expensive to perform [2, 3]. For this reason, the test is invariably substituted in epidemiological studies by simpler surrogate indexes including fasting insulin, the HOMA-IR index, the QUICKI or OGTT-based measures [8].

The prevalence of IR worldwide is increasing at an alarming rate, secondary to the obesity pandemic and decreasing levels of physical activity [9]. Thus, a critical need exists for more accurate diagnostic tests of insulin sensitivity that are able to detect those at greatest risk of adverse metabolic consequences of IR [10, 11]. Surrogate measures of IR possess suboptimal diagnostic sensitivity, especially among people without obesity, and are hampered by the lack of standardisation of the insulin assay [11, 12]. Diagnostic approaches leveraging blood-based signatures derived from the measurement of multiple biomarkers have shown promise and may allow for the more reliable identification of individuals at high cardiometabolic risk [13]. Here, we assess the utility of this approach in explaining the variability in insulin sensitivity as estimated by the *M* value using high-throughput plasma proteomics in two of the largest studies to date that have implemented the EIC: the Relationship between Insulin Sensitivity and Cardiovascular disease (RISC) [14] and the Uppsala Longitudinal Study of Adult Men (ULSAM) [15].

## Methods

### Study populations

The RISC and ULSAM studies are described extensively elsewhere [14, 15]. Briefly, RISC was a prospective observational cohort study of 1037 healthy people aged 30–60 years from 19 centres in 14 European countries whose main aim was to examine whether insulin sensitivity independently predicts CVD risk over 3–10 years’ follow-up [14].

The ULSAM study is an ongoing longitudinal epidemiological study based on all available men born between 1920 and 1924 and living in Uppsala County, Sweden [15]. The men were interviewed and examined at the ages of 50, 60, 70, 77, 82, 88 and 93 years, but the EIC was performed once at the age 70 visit in the early 1990s [6, 7].

### Measurement of protein biomarkers

We measured a total of 828 proteins in the plasma of RISC and ULSAM participants using the proximity extension assay (PEA) developed by Olink [16]. A detailed description is available in the electronic supplementary material (ESM Methods).

We used plasma obtained at the baseline visits between 2002 and 2004 for 1037 RISC participants and at the 1991–1995 visits for 954 ULSAM participants. For RISC, blood used for protein analysis was drawn within 1 month of the day of the EIC while, for ULSAM, blood was drawn on the same day as the EIC.

### Outcome measure and covariates

In each study, the *M* value, the rate of glucose disposal, was calculated as the amount of glucose taken up during the EIC study and was transformed to milligrams per kilogram body weight per minute. More details are provided in ESM Methods.

From the clinical databases of each study, we extracted covariates related to IR that were collected using standardised study protocols at the same time that the EIC was performed to include in our multivariable analyses, including age, sex (where applicable), BMI, systolic blood pressure (SBP) and standardised measures of cholesterol [4, 14].

### Statistical analyses

**Fig. 1:**
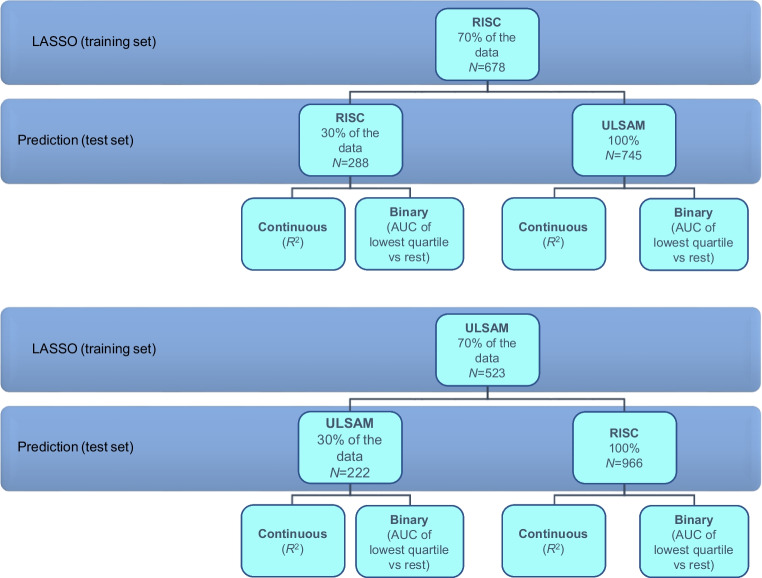
Flow chart showing the different data sources and design used in this study

Figure 1 summarises the different data sources used and our analytical approach. We applied the least absolute shrinkage and selection operator (LASSO) method to develop regression models incorporating multiple protein levels [17]. The *M* value was analysed as a continuous variable, and we also analysed IR as a binary variable, comparing people below the first quartile of the *M* value to the 75% above it. This quartile threshold has been previously linked to a sharp increase in adverse health outcomes related to IR [18]. In the RISC and ULSAM cohorts separately, models were trained on a randomly selected 70% of the cohort and tested on the remaining 30%. In addition, we assessed the portability of the RISC model to the ULSAM cohort and vice versa.

We applied LASSO regression in models that included three sets of clinical predictors: (1) recruitment centre, age, sex, BMI (basic clinical predictor model); (2) recruitment centre, age, sex, BMI, lipids, SBP (standard clinical predictor model); and (3) BMI, lipids and SBP or the subset of covariates whose distribution overlapped substantially across both cohorts (shared clinical predictor model). The lipids covariate was in form of the triglyceride to HDL-cholesterol ratio, which is arguably the simplest and most closely linked measure to IR [19]. For each of these sets of covariates, we also trained a model that additionally included proteins. A fourth protein-only model aimed to assess the performance of plasma proteins in the absence of any clinical covariates. Finally, we combined the HOMA-IR index with the standard covariates, again with and without proteins. As plasma insulin assays are not standardised, this final model was not tested across cohorts.

The standard clinical predictor model without and then with proteins was our a priori primary model of interest, as it establishes the added incremental value of protein measurements over and above all routinely available anthropomorphic and laboratory measurements for the assessment of IR in clinical practice. The basic clinical predictor model was formed by restricting the standard clinical predictor model to covariates believed to be causally associated with IR. We performed sensitivity analyses excluding proteins with a high proportion of measures flagged as being below the limit of detection (LOD). The standard clinical predictor model trained in ULSAM was also tested in the subset of men only in RISC. We generated predicted *M* values with our LASSO models and calculated standard diagnostic test statistics summarising the ability of the linear models to correctly classify whether an individual’s *M* value fell within the lowest quartile of measured *M* values.

In our final analysis we ran the randomised LASSO stability selection algorithm as presented by Meinshausen and Bühlmann [20] and implemented in R package stabs with the improved error bounds introduced by Shah and Samworth [21, 22]. We implemented this algorithm not only on the raw *M* values but also on residuals of the *M* values after progressively regressing out clinical covariates of interest, including centre (where applicable), age, sex (where applicable), BMI, lipids and blood pressure. We did not implement this algorithm on the binary *M* values. A detailed description is available in ESM Methods.

## Results

### Cohort characteristics

The baseline characteristics of the RISC and ULSAM participants included in the analyses are shown in Table 1. The cohort characteristics are fully described in ESM Results. Protein levels and distributions in the RISC and ULSAM cohorts using the relative Normalised Protein eXpression (NPX ) scale are shown in ESM Table 1.

**Table 1:** Characteristics of RISC and ULSAM participants at the time of EIC assessment

Characteristic	RISC (*N*=966)	ULSAM (*N*=745)
No. recruitment centres	22 (100)	1 (100)
Male	434 (45)	745 (100)
Age (years)	44.5 (8.3)	71.0 (0.59)
BMI (kg/m^2^)	25.4 (4.0)	26.0 (3.18)
Blood pressure (mmHg)		
Systolic	118 (12.2)	146 (19.6)
Diastolic	75 (7.8)	84 (11.3)
Blood cholesterol (mmol/l)		
Triglycerides	1.11 (0.75)	1.38 (0.66)
HDL-C	1.43 (0.38)	1.30 (0.35)
LDL-C	2.93 (0.79)	3.92 (0.90)
Total cholesterol	4.86 (0.87)	5.84 (1.0)
Triglyceride/HDL-C ratio	2.10 (2.51)	1.20 (0.83)
HOMA-IR	1.92 (0.57)	0.94 (0.57)
EIC		
*M* value, continuous	7.12 (2.95)	5.45 (1.97)
*M* value, no. insulin resistant^a^	242 (25)	186 (25)

Data are *n* (%) or mean (SD)

^a^Threshold cut-off for IR is the lowest quartile

HDL-C, blood HDL-cholesterol concentration; LDL-C, blood LDL-cholesterol concentration

### Standard linear regression analyses and replication of marginal effects of proteins

A total of 359 and 317 proteins for RISC and ULSAM, respectively, were significant at a false discovery rate of <0.05 in the single protein association tests including age, sex and centre covariates. When BMI was also included, these numbers decreased to 271 and 241, respectively. The number of overlapping proteins between the two cohorts was 168 and 72 for the two sets of covariates, respectively. Specifically, among the significant proteins in RISC, 46.8% (168/359) and 26.6% (72/271) were replicated in ULSAM and, among the significant proteins in ULSAM, 53.0% (168/317) and 29.9% (72/241) were replicated in RISC. Corresponding statistics using a more stringent Bonferroni correction are provided in ESM Results. Full results are shown in ESM Table 2.

### LASSO regression models

**Fig. 2:**
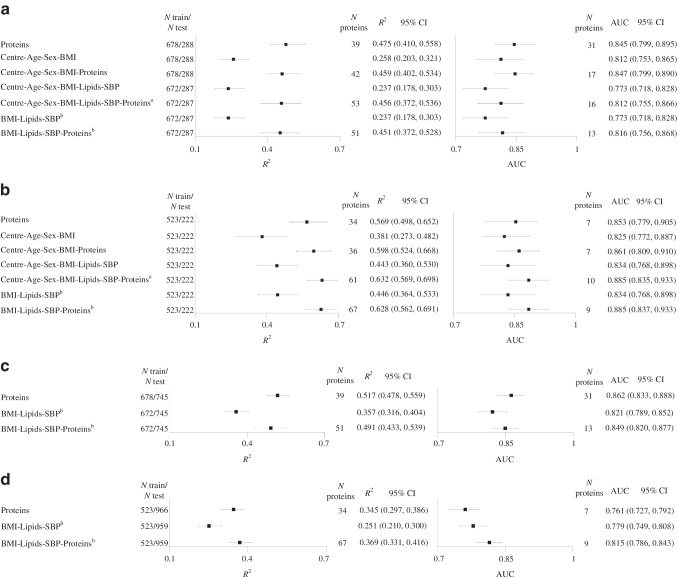
Variance explained (*R*^2^) using *M* as a continuous variable, and the AUC statistic using *M* as a binary variable. (**a**, **b**) Models performed using the training and the test datasets from the same cohort: (**a**) RISC and (**b**) ULSAM. (**c**, **d**) Models performed using the training dataset from one cohort and the testing dataset from the other cohort: (**c**) RISC vs ULSAM and (**d**) ULSAM vs RISC. In ULSAM, age is a limited covariate (69–73 years) and the variables centre and sex are invariant as there was only one centre and all participants were males. ^a^A priori main model. ^b^Models with common covariates in both cohorts. BMI was selected in all of the only-covariates models performed. Lipids were additionally selected in the only-covariates models in ULSAM only when *M* was used as a continuous variable

Consistent with previous reports [23, 24], standard clinical variables alone (age, sex, centre, BMI, lipids and SBP) explained 0.237–0.258 of the *M* value *R*^2^ in RISC and 0.381–0.446 of the *M* value variance in ULSAM when validation was performed within the same cohort (Fig. 2a,b). LASSO regression models selected 39–53 proteins in RISC and 34–67 proteins in ULSAM. Compared with covariate models alone, protein models resulted in absolute increases of *R*^2^ ranging from 0.201 to 0.238 for RISC and from 0.123 to 0.217 for ULSAM. Importantly, the 95% CIs of *R*^2^ for a model including proteins did not overlap the 95% CIs for the corresponding model without proteins. Models with proteins alone explained a similar proportion of *M* value variance to models with proteins and clinical variables. Specifically for our primary model of interest, A standard LASSO model incorporating 53 proteins in addition to routinely available clinical variables increased the *M* value *R*^2^ from 0.237 (95% CI 0.178, 0.303) to 0.456 (0.372, 0.536) in RISC and from 0.443 (0.360, 0.530) to 0.632 (0.569, 0.698) in ULSAM with the addition of 61 proteins. Findings for the binary IR variable generally mirrored those for the continuous *M* value. Adding proteins to a set of covariates increased the AUC by 0.035–0.072 for RISC and by 0.036–0.051 for ULSAM, but the 95% CIs had a substantial amount of overlap.

We observed a similar but less marked degree of improvement in the proportion of the *M* value variance explained by the addition of proteins when we trained models in one cohort and tested them in the other cohort (Fig. 2c,d). Standard clinical variables alone (BMI, lipids and SBP) explained 0.357 of *M* value variance in ULSAM, and 0.251 of *M* value variance in RISC. Proteins alone led to an increase of 0.160 over the model of shared covariates alone and 0.134 when combined with shared covariates in ULSAM (Fig. 2c), while the respective increases in RISC were 0.118 and 0.094 (Fig. 2d). As in the within-cohort analyses, the 95% CIs of *R*^2^ for a model including proteins did not overlap the 95% CIs for the corresponding model without proteins.

Findings for the within and cross-cohort analyses of the binary IR variable generally mirrored those for the continuous *M* value, with absolute improvements in the AUC observed in the models incorporating proteins of 0.028–0.041 over clinical predictor models (Fig. 2a–c). The one exception was a 0.018 reduction in the AUC when comparing the protein-only model with the shared covariate model ported from ULSAM to RISC (Fig. 2d). However, 95% CIs for absolute measures of AUC showed substantial overlap between corresponding models. Restricting the testing cohort to male participants in RISC did not improve performance of the model trained on the male-only ULSAM cohort (ESM Table 3).

The results of LASSO models that included HOMA-IR as an additional clinical variable increased *R*^2^ in RISC from 0.237 to 0.330 (ESM Table 4). However, no appreciable increase in *R*^2^ was observed in ULSAM. When tested within RISC, LASSO models selected HOMA-IR and BMI, which together explained 0.330 of *M* value variance, while BMI, lipids and HOMA-IR explained 0.430 of *M* value variance in ULSAM. Adding proteins as covariates nevertheless increased *R*^2^ by 0.139 in RISC and 0.136 in ULSAM, an increase comparable to that seen in models that did not include HOMA-IR.

Comparing predicted vs actual *M* value classifications using our linear predictor we observed an increase of ~10% to 25% for sensitivity and ~7% to 15% for balanced accuracy across the various models that included proteins compared with analogous models restricted to clinical predictors alone (Table 2). Full cross-tabulations of observed and predicted *M* values by class (lowest quartile = case, rest = non-case) as well as additional diagnostic test proportions are provided in ESM Table 5.

**Table 2:** Selected diagnostic test characteristics for all linear models to detect an *M* value in the lowest quartile

Model covariates	Sensitivity	Specificity	PPV	NPV	Balanced accuracy
RISC → RISC					
Proteins	0.458	0.963	0.805	0.842	0.711
Centre-Age-Sex-BMI	0.208	0.986	0.833	0.789	0.597
Centre-Age-Sex-BMI-Proteins	0.542	0.912	0.672	0.857	0.727
Centre-Age-Sex-BMI-Lipids-SBP	0.250	0.981	0.818	0.796	0.616
Centre-Age-Sex-BMI-Lipids-SBP-Proteins	0.444	0.916	0.640	0.831	0.680
BMI-Lipids-SBP	0.236	0.981	0.810	0.793	0.609
BMI-Lipids-SBP-Proteins	0.486	0.930	0.700	0.844	0.708
ULSAM → ULSAM					
Proteins	0.250	0.964	0.700	0.792	0.607
Centre-Age-Sex-BMI	0.107	1.000	1.000	0.769	0.554
Centre-Age-Sex-BMI-Proteins	0.357	0.976	0.833	0.818	0.667
Centre-Age-Sex-BMI-Lipids-SBP	0.145	0.994	0.889	0.778	0.570
Centre-Age-Sex-BMI-Lipids-SBP-Proteins	0.455	0.988	0.926	0.845	0.721
BMI-Lipids-SBP	0.164	0.994	0.900	0.782	0.579
BMI-Lipids-SBP-Proteins	0.455	0.988	0.926	0.845	0.721
RISC → ULSAM					
Proteins	0.299	0.984	0.862	0.807	0.642
BMI-Lipids-SBP	0.075	0.996	0.875	0.764	0.536
BMI-Lipids-SBP-Proteins	0.226	0.986	0.840	0.793	0.606
ULSAM → RISC					
Proteins	0.310	0.942	0.641	0.803	0.626
BMI-Lipids-SBP	0.213	0.969	0.699	0.787	0.591
BMI-Lipids-SBP-Proteins	0.479	0.915	0.653	0.840	0.697

Sensitivity = TP/(TP+FN); specificity = TN/(TN+FP); PPV = (sensitivity×prevalence)/((sensitivity×prevalence)+((1–specificity)×(1–prevalence))); NPV = (specificity×(1–prevalence))/(((1–sensitivity)×prevalence)+((specificity)×(1–prevalence))); balanced accuracy = (sensitivity+specificity)/2

FN, false negative; FP, false positive; NPV, negative predictive value; PPV, positive predictive value; TN, true negative; TP, true positive

RISC → RISC, models trained in RISC and tested in RISC; ULSAM → ULSAM, models trained in ULSAM and tested in ULSAM; RISC → ULSAM, models trained in RISC and tested in ULSAM; ULSAM → RISC, models trained in ULSAM and tested in RISC

In sensitivity analyses, excluding proteins flagged as being below the LOD using three different LOD cut-offs (25%, 10% and 3%) gave similar *R*^2^ and AUC results to the main analyses (ESM Table 6).

The cross-validation mean squared error plots, including upper and lower SE bars, along the λ sequence for the RISC and ULSAM cohorts are shown in ESM Figs 1–4. The root mean squared errors (RMSEs) corresponding to each LASSO regression model obtained are shown in ESM Fig. 5.

### Proteins consistently selected by standard LASSO and randomised LASSO stability selection analyses

ESM Tables 7–9 list the proteins selected by standard LASSO analyses for each model run, the number of times a protein was selected among a set of models, and the correlation among proteins selected and not selected by LASSO. A total of 135 proteins were selected by LASSO in one or more models. Six proteins were selected in ten or more of the 16 main LASSO models run. These were insulin-like growth factor-binding protein 2 (IGFBP2), leptin (LEP), reticulon-4 receptor (RTN4R), adhesion G protein-coupled receptor G1 (ADGRG1), inhibin beta C chain (INHBC) and lipoprotein lipase (LPL). While selected proteins were less correlated than non-selected proteins, ~25% to 30% of pairwise correlations still had an *r*>0.2 compared with 30–40% among non-selected proteins.

Our randomised LASSO stability selection analyses selected subgroups of ten proteins in RISC and seven proteins in ULSAM in up to six *M* value models tested per cohort (Table 3). Four proteins were selected in both cohorts: fatty acid-binding protein 4 (FABP4), IGFBP2, LEP and RTN4R. The number of proteins selected decreased progressively as more covariates were regressed out of the *M* value, with only two proteins selected for fully regressed models. IGFBP2 was the only protein selected in all models in both cohorts. Improvements in *R*^2^ for stability selection proteins were intermediate to those observed with standard LASSO regression models in both RISC (range 0.352–0.366) and ULSAM (range 0.472–0.587) but still substantially higher than in models restricted to covariates alone. *R*^2^ for cross-cohort analyses remained largely similar in both directions (RISC to ULSAM range 0.444–0.524; ULSAM to RISC range 0.345–0.348) to *R*^2^ for standard LASSO models (RISC to ULSAM range 0.491–0.517; ULSAM to RISC range 0.345–0.369).

**Table 3:** Proteins selected by randomised LASSO stability selection analyses when regressed on raw *M* values or on the *M* value residuals after regressing out clinical predictors of insulin sensitivity

Covariates regressed out of *M* value	None	Centre*-Age	Centre*-Age-Sex*	Centre*-Age-Sex*-BMI	Centre*-Age-Sex*-BMI-Lipids-SBP	BMI-Lipids-SBP
Protein selected in RISC						
ADGRG2_Q8IZP9_metabo	x	x				
CD300LG_Q6UXG3_develo				x		
FABP2_P12104_cardio_ii			x			
FABP4_P15090_cardio_iii	x	x	x			
IGFBP_1_P08833_cardio_iii	x	x	x			
IGFBP_2_P18065_cardio_iii	x	x	x	x	x	x
INHBC_P55103_develo	x	x	x			
LEP_P41159_cardio_ii	x	x	x			
RTN4R_Q9BZR6_metabo	x	x	x			
SCGB3A2_Q96PL1_cardio_iii	x	x	x	x	x	x
Protein selected in ULSAM						
ADGRG1_Q9Y653_organ_damage	x	x	N/A			
FABP4_P15090_cardio_iii	x		N/A			
IGFBP_2_P18065_cardio_iii	x	x	N/A	x	x	x
ITGAV_P06756_oncolo_ii	x	x	N/A	x	x	x
LEP_P41159_cardio_ii	x	x	N/A			
LPL_P06858_cardio_ii	x	x	N/A	x		
RTN4R_Q9BZR6_metabo		x	N/A			
Variance (*R*^2^) explained by proteins selected (95% CI)						
RISC **→** RISC	0.366	0.363	0.381	0.338	0.362	0.352
	(0.281–0.452)	(0.278–0.448)	(0.297–0.465)	(0.253–0.425)	(0.277–0.448)	(0.266–0.439)
ULSAM **→** RISC	0.348					0.345
	(0.300–0.396)					(0.296–0.393)
ULSAM **→** ULSAM	0.582	0.587		0.472	0.495	0.495
	(0.502–0.663)	(0.507–0.667)		(0.380–0.565)	(0.406–0.586)	(0.404–0.585)
RISC **→** ULSAM	0.524					0.444
	(0.475–0.573)					(0.391–0.497)

^*^Variable not applicable in ULSAM cohort.

Protein label includes name, UniProt ID, panel

*R*^2^ = variance explained of *M* value of selected proteins and covariates in the testing dataset. Numbers in parentheses provide 95% confidence intervals for given *R*^2^.

N/A, not applicable as ULSAM included only male participants.

RISC → RISC, models trained in RISC and tested in RISC; ULSAM → ULSAM, models trained in ULSAM and tested in ULSAM; RISC → ULSAM, models trained in RISC and tested in ULSAM; ULSAM → RISC, models trained in ULSAM and tested in RISC

## Discussion

We aimed to develop a novel blood-based proteomic signature to reliably estimate a direct measure of insulin sensitivity in men and women with normoglycaemia or impaired glucose tolerance using a high-throughput platform able to measure reliably hundreds of low-abundance proteins in plasma. Our principal findings are fourfold.

First, a large proportion of the 823 proteins measured with the PEA platform were statistically associated with IR in both the RISC and the ULSAM cohorts. Approximately half of the significant protein associations in one cohort were replicated in the other cohort and vice versa. While such observations may, in part, be driven by the correlation structure of the proteins measured in plasma, we noted several well-established proteins among the significant associations, as well as several novel ones. These results serve as a compelling starting point for further inquiry into the role of these proteins in the pathophysiology of IR.

Second, protein models derived by standard LASSO regression of the *M* value explained a notably larger *R*^2^ than models restricted to commonly available clinical variables related to IR. This increase was most evident when applying the standard LASSO within each cohort but was also present when the model from one cohort was applied to the other cohort, despite dramatic differences between the two in the distribution of anthropometric characteristics and health status at the time of the clamp. The increased variance explained persisted even in the face of an increasing degree of stringency in the threshold for inclusion of proteins with values below the LOD, suggesting substantial redundancy in the information provided by measuring hundreds of proteins in the plasma. While patterns were consistent when the *M* value was transformed into a binary indicator of IR, 95% CIs for AUCs overlapped substantially, probably reflecting a loss of power from discretising the outcome, as well as suboptimal inference for testing the null hypothesis of a delta AUC not equal to zero [25].

Third, a substantially higher proportion of the *R*^2^ of the *M* value was explained by proteins alone than by clinical variables alone in each cohort, even after including HOMA-IR among the clinical variables. Furthermore, a protein-only model performed as well as models that combined clinical variables and proteins together in most cases. These findings suggest that plasma protein signatures for IR derived from the proteins we measured not only provide incremental value to clinical variables but can also replace predictive clinical variables when they are not available. Consistent with this hypothesis is the observation that ~25% of proteins significantly associated with the *M* value when adjusting for age and sex alone lost their significance when BMI was added as a covariate.

Fourth, a stability selection algorithm including randomised LASSO selected a substantially smaller number of proteins and reduced the proportion of *R*^2^ explained within a cohort to an intermediate degree between that observed with clinical variables alone and that observed with clinical variables combined with the proteins selected by a standard LASSO approach. Differences in *R*^2^ were less marked for cross-cohort analyses. In these randomised LASSO cross-cohort analyses, models including only a small number of stable proteins achieved nearly the same performance as the standard LASSO cross-cohort analyses. For models trained in RISC and tested in ULSAM, the two ‘stable’ proteins in addition to clinical covariates achieved an *R*^2^ of 0.444, while the 51 standard LASSO proteins achieved an *R*^2^ of 0.491. Similarly, for models trained in ULSAM and tested in RISC, two ‘stable’ proteins achieved an *R*^2^ of 0.345, while 67 standard LASSO proteins achieved an *R*^2^ of 0.369. These findings suggest the presence of unstable features leading to some degree of model overfitting within a cohort despite our use of testing and training sets and cross-validations. They also suggest that proteins selected by the randomised LASSO method are likely to be the most generalisable and robust if implemented clinically to help predict IR across multiple populations, even when the baseline characteristics of those populations are quite divergent.

Few studies have reported on the utility of high-throughput proteomics of plasma to more reliably estimate IR [13]. Several studies have focused on the identification of prevalent type 2 diabetes or the prediction of incident type 2 diabetes while others have examined surrogate measures of IR [13]. To the best of our knowledge, this is the first study to combine high-throughput methodology with a direct measure of insulin sensitivity. Our findings highlight the potential of using a proteomic signature to estimate a direct measure of insulin sensitivity over and above the use of clinical variables and across a wide range of age and baseline health states. Such analyses may provide opportunities to markedly improve the identification of insulin-resistant individuals at risk of IR-related adverse health consequences [1, 10].

Several proteins selected repeatedly by LASSO for both continuous and binary outcomes in our primary analyses have long-standing and well-established connections to IR. The two most frequently selected included IGFBP2 and LEP, which also possessed the largest multivariate effect sizes per SD increase in measured protein. IGFBP2 is associated with multiple cardiovascular risk factors related to the metabolic syndrome and IR and is further regulated by LEP [26–29]. LEP regulates food intake, body weight and glucose metabolism, is associated with IR independent of body fat mass, and lowers blood glucose and insulin even in the absence of weight loss in mice [30]. LPL, the third most frequently selected protein, is also associated with obesity and other metabolic disorders related to energy balance, insulin action and body weight regulation [31]. More specifically, LPL is an important enzyme in the metabolism of triacylglycerol-rich lipoproteins associated with IR, including triglycerides, chylomicrons and very low-density lipoproteins. By facilitating adhesion of these lipoproteins to the vascular endothelium of specific tissues, LPL plays an important role in their targeted delivery to and use by insulin-sensitive tissues, such as muscle and liver, and their clearance from the blood [31]. Overexpression of LPL in target tissues such as muscle causes muscle-specific IR by promoting dysfunction in insulin signalling and action [32]. These three proteins were also selected by our stability selection algorithm.

Three proteins selected frequently by LASSO with less established roles in IR include RTN4R, ADGRG1 and INHBC. RTN4R has important roles in regulating axon regeneration and neuronal plasticity in the central nervous system, but the canonical ligand for RTN4R (reticulin 4) is also expressed in non-parenchymal cells within the liver where it blocks diet-induced hepatic lipid accumulation, steatosis and IR [33]. ADGRG1 is a receptor that facilitates adhesion of cells to collagen matrix, especially in the developing brain, but it is also the most highly expressed G protein-coupled receptor in human and mouse pancreatic islets, with a high correlation between expression and many genes essential for beta cell function [34]. Lastly, INHBC is a member of the TGF-β family involved in the regulation of the secretion of follicle-stimulating hormone by the pituitary gland along with other inhibins and activins, and thus regulates hypothalamic and pituitary hormone secretion as well as the secretion of gonadal hormones and insulin [35, 36]. The *INHBC* gene is also highly expressed in the liver [37]. These three proteins were also selected by our stability selection algorithm. Two additional proteins with no obvious prior link to IR, secretoglobin family 3A member 2 (SCGB3A2) and integrin subunit alpha V (ITGAV), were selected by all stability selection algorithm models for RISC and ULSAM, respectively, and are worth highlighting. SCGB3A2 is a small secretory protein that is predominantly expressed in airway epithelial Club cells, It has anti-inflammatory, growth factor, anti-fibrotic and anti-cancer activities that influence various lung diseases including asthma [38]. ITGAV may regulate angiogenesis and cancer progression [39].

A key strength of our study is the profiling of many low-abundance proteins in plasma from two of the largest studies conducted to date that have made direct measurements of insulin sensitivity using the reference standard, the EIC, as well as undertaking comprehensive and standardised documentation of clinical risk factors related to IR. Our study is therefore the most comprehensive plasma proteomics study to date on IR. We note that the absolute incremental improvement in variance explained by the proteomic signature was comparable in the two cohorts despite the vastly different cardiometabolic risk profiles at baseline. This large difference in baseline health state in the two cohorts is likely to be responsible for the substantial differences in the proportion of variance explained by the clinical variables alone.

Our study also has weaknesses that are worth noting. First, the plasma used for the RISC study was not collected on the day of the EIC but rather within 1 month of the EIC. However, a recent study demonstrated remarkable stability and reproducibility of most protein measurements using the PEA over a period of 1 year [40]. Furthermore, any misclassification related to biological variability is likely to be non-differential and bias our results towards the null. Second, we did not assess the entire plasma proteome. Our limited assessment was also not completely unbiased as several of the panels we measured were designed specifically for the study of cardiometabolic disease. This semi-targeted platform thus might limit the utility of additional proteins, but this assumes that knowledge of predictors is already saturated, which is unlikely given that at least 5000 additional canonical and non-redundant proteins have been catalogued in the Human Plasma PeptideAtlas build 2021-07 [41]. Third, we were limited in our ability to demonstrate portability of the proteomic signature to people of non-European race and ethnicity with a differing prevalence of and predisposition to IR. Thus, further study in these populations is necessary to ensure that implementation of this approach does not further promote health disparities. Fourth, the cost of complete proteomic profiling remains very high, but the availability of custom panels has the potential to cut costs and facilitate the clinical implementation of this technology. Lastly, both the RISC and ULSAM studies are too restricted in their size and follow-up to have the power to clearly demonstrate a benefit of the *M* value in predicting adverse outcomes over modestly correlated proxy measures. By extension, this limitation also applies to the assessment of the incremental benefit of plasma proteomics. However, the relative utility of proteomic signatures for *M* values vs proxies of IR could soon be tested in large-scale population studies such as UK Biobank using the same technology [42].

In summary, our results suggest that plasma proteomic profiling has the potential to improve individual assessments of insulin sensitivity based on a reference measure. The measurement of additional proteins in combination with other -omics profiling should be explored to determine whether an even more accurate estimation of an individual’s insulin-mediated glucose disposal/uptake and subsequent risk of health consequences can be made and whether this approach can be successfully implemented in clinical practice.

## Supplementary Information

Below is the link to the electronic supplementary material.

Supplementary file1 (PDF 961 KB)

Supplementary file2 (XLSX 417 KB)

## Data Availability

Association results for all plasma proteins measured in both cohorts are provided in the ESM. Qualified researchers are welcome to submit proposals for collaborative work in either ULSAM (https://www.pubcare.uu.se/ulsam/Research/Proposals) or RISC (http://www.egir.org/index.html). Such collaborative work could include access to individual-level data on approval of the research proposal.

## Abbreviations

ADGRG1: Adhesion G protein-coupled receptor G1
ASCVD: Atherosclerotic CVD
EIC: Euglycaemic–hyperinsulinaemic clamp
FABP4: Fatty acid-binding protein 4
IGFBP2: Insulin-like growth factor-binding protein 2
INHBC: Inhibin beta C chain
IR: Insulin resistance
ITGAV: Integrin subunit alpha V
LASSO: Least absolute shrinkage and selection operator
LEP: Leptin
LOD: Limit of detection
LPL: Lipoprotein lipase
PEA: Proximity extension assay
RISC: Relationship between Insulin Sensitivity and Cardiovascular disease
RTN4R: Reticulon-4 receptor
SBP: Systolic blood pressure
SCGB3A2: Secretoglobin family 3A member 2
ULSAM: Uppsala Longitudinal Study of Adult Men
