# Supplementary material for: Plasma proteomic signatures of a direct measure of insulin sensitivity in two population cohorts
Source: Diabetologia. 2023 Jun 17;66(9):1643–54. doi: 10.1007/s00125-023-05946-z (PMC10390625; doi:10.1007/s00125-023-05946-z)
Supplement: Supplementary file 1 — Supplementary file1 (PDF 961 KB) [file 125_2023_5946_MOESM1_ESM.pdf]

Electronic supplementary material (ESM)

Plasma proteomic signatures of a direct measure of insulin sensitivity in two population cohorts

Zanetti et al.

ESM Methods

Measurement of Protein Biomarkers

The proteins measured were from nine OLINK® Target panels each focused around disease areas or biologic processes including Cardio-metabolic, Cardiovascular II, Cardiovascular III, Development, Immune response, Inflammations, Metabolism, Oncology II and Organ damage panels. Each panel includes 92 proteins including a smaller set of well-known and established markers combined with a larger set of more exploratory markers for novel discovery (http://www.olink.com/proseekmultiplex/complete-biomarker-list/). A small number of proteins were present on more than one panel (e.g., IL-6).

Real-time quantitative polymerase chain reaction (qPCR) is used in the readout step to measure relative changes in protein expression. The qPCR detects the unique DNA sequence formed when complementary oligonucleotide-tags attached to pairs of analyte-specific antibodies hybridize and extend in the presence of DNA polymerase. OLINK® translates the Ct values from the qPCR into the relative quantification unit, Normalized Protein eXpression (NPX), using a series of computations. OLINK® translates the Ct values from the qPCR into the relative quantification unit, Normalized Protein eXpression (NPX), using the following equations:

Extension Control: $Ct_{Analyte} - Ct_{Extension\ Control} = dCt_{Analyte}$

Inter-plate Control: $dCt_{Analyte} - dCt_{Inter\text{-}plate\ Control} = ddCt_{Analyte}$

Adjustment against a correction factor: $\text{Correction factor} - ddCt_{Analyte} = NPX_{Analyte}$

The NPX is a relative quantification unit logarithmically related to protein concentration, where a high value corresponds to a higher protein expression. NPX data allows users to identify changes for individual protein levels across their sample set, and then use this data to establish protein signatures (https://www.olink.com/faq/how-is-the-data-pre-processed/).

Outcome measure and covariates

In each study, M-value, the total amount of glucose infused during the steady-state period of the euglycemic hyperinsulinemic clamp (EIC) procedure, served as a measure of the subject's sensitivity to the prevailing plasma insulin concentrations with higher M-values being consistent with more insulin sensitivity.

RISC: RISC participants had 2 baseline examination visits if they met initial inclusion and exclusion criterion. At the first visit they underwent a 75-g oral glucose tolerance test (OGTT) and if they then met the second set of exclusion criterion, they were scheduled for the Euglycemic clamp on a separate day within 1 month of the

OGTT. We used plasma samples collected, aliquoted, and stored from the OGTT study day visit for the proteomic measurements, which was also when the clinical phenotypic data was collected through multiple questionnaires and a physical exam was performed to document height, weight, waist/hip/thigh circumferences, bioimpedance estimate of percent fat/fat-free mass using TANITA scale, heart rate and blood pressure. During the EIC, the target plasma glucose concentration was maintained between 4.5 and 5.5 mmol/l and insulin infused at a rate of 240 $pmol \cdot min^{-1} \cdot m^{-2}$. Bedside plasma (or blood) glucose was measured at 5- to 10-min intervals to ensure it remains within 0.8 mmol/l (±15%) of the target glucose concentration. The steady-state period was between 80 to 120 minutes.

ULSAM: For ULSAM, participants followed similar procedures as in RISC but in the context of a return visit for their 20 years follow up exam at the age of approximately 70 years (cohort started when all participants were age 50). The OGTT and the clamp procedure were performed at least 1 week apart. The plasma samples used to measure OLINK proteins were from the day of the clamp procedure. The euglycemic insulin clamp in this cohort was performed with a target plasma glucose concentration of 5.1 mmol/L. The infusion rate of insulin was of 56 mU per minute per square meter of body surface. Bedside plasma glucose was measured at 5-min intervals to ensure it remained within +/- 0.2 mmol/l of the target glucose concentration. The M-value was calculated between 60 minutes and 120 minutes of infusion. In a subset of 17 men, replicate measurements to estimate measurement errors were performed[1]. The coefficient of variation for the M-value was 9.3%.
We applied an inverse-normal transformation to each protein within each cohort.

Statistical Analyses

We excluded subjects who failed sample quality control or were missing M-values in both cohorts. For ULSAM, we also excluded subjects with prevalent diabetes at the time of their age 70 clinic visit when they underwent the euglycemic clamp. We did not exclude ULSAM participants with prevalent cardiovascular disease at the time of their clamp. We then excluded proteins with a high proportion of missing measurements in either cohort. Sample evaluation was carried out on each plate and a sample plate median value was calculated for the Incubation Control 2 and the Detection Control, respectively. For each sample, the result for each of these internal controls was allowed to deviate no more than Â±0.3NPX from the plate median. If any or both internal controls exceeded the 0.3 NPX limit, the sample failed the QC. If more than 1/6th of the samples failed the QC, the run was deemed unreliable. The reason for the issues was then be evaluated and (if applicable) samples were rerun (https://olink.com/faq/how-is-quality-control-of-the-data-performed/).

We first performed standard linear regression in the RISC and ULSAM cohorts independently to calculate marginal associations of the M-value with each of the OLINK proteins measured. All association analyses included covariates age, sex, and recruitment center. Then, we ran a second model additionally including body mass index (BMI). The Benjamini-Hochberg False Discovery Rate (FDR) method and a

Bonferroni-corrected alpha threshold (adjusting for the final 823 proteins analyzed) were used to identify significant associations.

To choose the LASSO regularization parameter lambda (λ), we used the *cv.glmnet* function in glment to perform cross validations over a total of 10 folds. The hyperparameter tuning was done using the function glment at alpha=1 (lasso penalty) and applying the one-standard-error rule, using the value of λ that gave the most regularized model whose cross-validation error was within one standard error of the minimum cross-validation error (lambda = lambda.1se). In the RISC and ULSAM cohorts separately, models were trained on a randomly selected 70% of the cohort and tested on the remaining 30%. The split into training/testing was done using the *createDataPartition* function in R using as a reference (a vector of outcomes=y) the M value to have in all the data set created (training and test) the same proportion of cases and controls (1:3) and to avoid any type of case number imbalances.

Our final analysis included a stability selection algorithm to improve the selection process of proteins and to obtain an error control for the number of falsely selected noise variables. This algorithm extends the standard LASSO approach to perform a LASSO regression multiple times on subsamples of the training data and returns a selection probability for each predictor (number of times selected divided by number of regressions done). This type of regularization has advantages in cases where the number of predictors exceeds the number of observations, in selecting variables consistently, demonstrating better error control and not depending strongly on the penalization parameter.

Lasso analyses were conducted with the R software, version 3.3.0 including the *glmnet and caret (confusionMatrix function)* packages and the *randLassoStabSel* package to perform the Randomized Lasso Stability Selection. The function uses the '*stabsel*' function from the 'stabs' package but implements the randomized lasso version. We followed the transparent reporting of a multivariable prediction model for individual prognosis or diagnosis (TRIPOD) statement.

ESM Results

<u>*Cohort Characteristics*</u>

Plasma protein profiling was attempted in 1037 subjects of the RISC study and all but 64 passed sample QC. We then further excluded 7 subjects with missing M-values leaving 966 subjects available for analysis. For ULSAM, profiling was attempted in 954 subject and all but 48 passed sample QC. We then further excluded 54 subjects with missing M-values and 107 subjects with prevalent diabetes leaving 745 subjects available for analysis.

We excluded five of the 828 proteins due to a high proportion (> 25%) of missing values in either cohort. Among the remaining proteins, no imputation of values was necessary as no subjects had missing

proteins levels. Furthermore, no imputation of clinical variables was necessary as only a very small number of subjects with full protein profiles were missing one or more covariates (n = 7 in RISC).

Of note, no overlap in the range of age exists between the two cohorts with a mean age of 44.4 years (SD, 8.3 years) in RISC at baseline and 70.9 years (SD, 0.6 years) in ULSAM. In RISC, 55% of participants were women while in ULSAM all participants were men.

Standard linear regression analyses and replication of marginal effects of proteins

We identified 141 and 136 proteins in RISC and ULSAM, respectively, passing the Bonferroni correction threshold of significance (alpha= $6.1*10^{-5}$ considering 823 proteins tested) for the first model adjusted for age, sex, and center. In the second model additionally adjusted for BMI, these numbers decreased to 69 and 72. Among significant proteins in RISC, 69/141 (48.9%) and 14/69 (20.3%) replicated in ULSAM for the first and second models respectively. Among significant protein in ULSAM, 69/136 (50.7%) and 14/72 (19.4%) replicated in RISC for the first and second models respectively.

References

[1] Berglund L, Berne C, Svardsudd K, Garmo H, Zethelius B (2009) Early insulin response and insulin sensitivity are equally important as predictors of glucose tolerance after correction for measurement errors. Diabetes Res Clin Pract 86(3): 219-224. 10.1016/j.diabres.2009.09.016

ESM Table 1. Proteins distributions in the Relationship between Insulin Sensitivity and Cardiovascular disease (RISC) and Uppsala Longitudinal Study of Adult Men (ULSAM) cohorts.

ESM Table 2. Age, sex, and recruitment center adjusted linear regression results of M value on each plasma protein in the Relationship between Insulin Sensitivity and Cardiovascular disease (RISC) and Uppsala Longitudinal Study of Adult Men (ULSAM) cohorts, before and after further adjustment for BMI.

ESM Table 3. Variance explained in the RISC dataset using the ULSAM trained models.

ESM Table 4. Variance explained (R2) using M as a continuous variable and area under the ROC Curve (AUC) statistic using M as a binary variable including the Homeostatic Model Assessment for Insulin Resistance (HOMA-IR) index in the LASSO regression models.

ESM Table 5. Full cross-tabulations of observed and predicted M-values by class (lowest quartile = case, rest = non-case) and additional diagnostic tests proportions.

ESM Table 6. Variance explained (R2) using M as a continuous variable and area under the ROC Curve (AUC) statistic using M as a binary variable excluding proteins with values below the lower limit of detection (LOD) at progressively more stringent cutoffs for removal of proteins.

ESM Table 7. Proteins selected by LASSO and their LASSO derived multivariable effect by linear (for continuous M-value) or logistic (for binary M-value) regression for all models tested in the Relationship between Insulin Sensitivity and Cardiovascular disease (RISC) and Uppsala Longitudinal Study of Adult Men (ULSAM) cohorts. Positive effect reflects the mean increase in M value per standard deviation (SD) increase in the level of the protein measured in the plasma, consistent with improved insulin sensitivity. A negative effect is consistent with a mean decrease in M value and insulin sensitivity.

ESM Table 8. Number of times a protein was selected among the named set of models.

ESM Table 9. Proportion of pairwise correlations of proteins reaching specified thresholds of r (correlation) >0.2, >0.4, >0.7, >0.9, stratified by proteins selected by at least one LASSO model (n = 135) versus not selected by LASSO (n = 638) and compared to all proteins together (823).

ESM Figure 1. The cross-validation curve plot with upper and lower standard deviation curves along the λ sequence (error bars) in the RISC cohort using M as a continuous variable. Model 1 performed only with proteins; Model 2 performed with proteins and age, sex, center, and body mass index; Model 3 performed with proteins and age, sex, center, body mass index, lipids, and systolic blood pressure; Model 4 performed with proteins and common clinical covariates: body mass index, lipids, and systolic blood pressure.

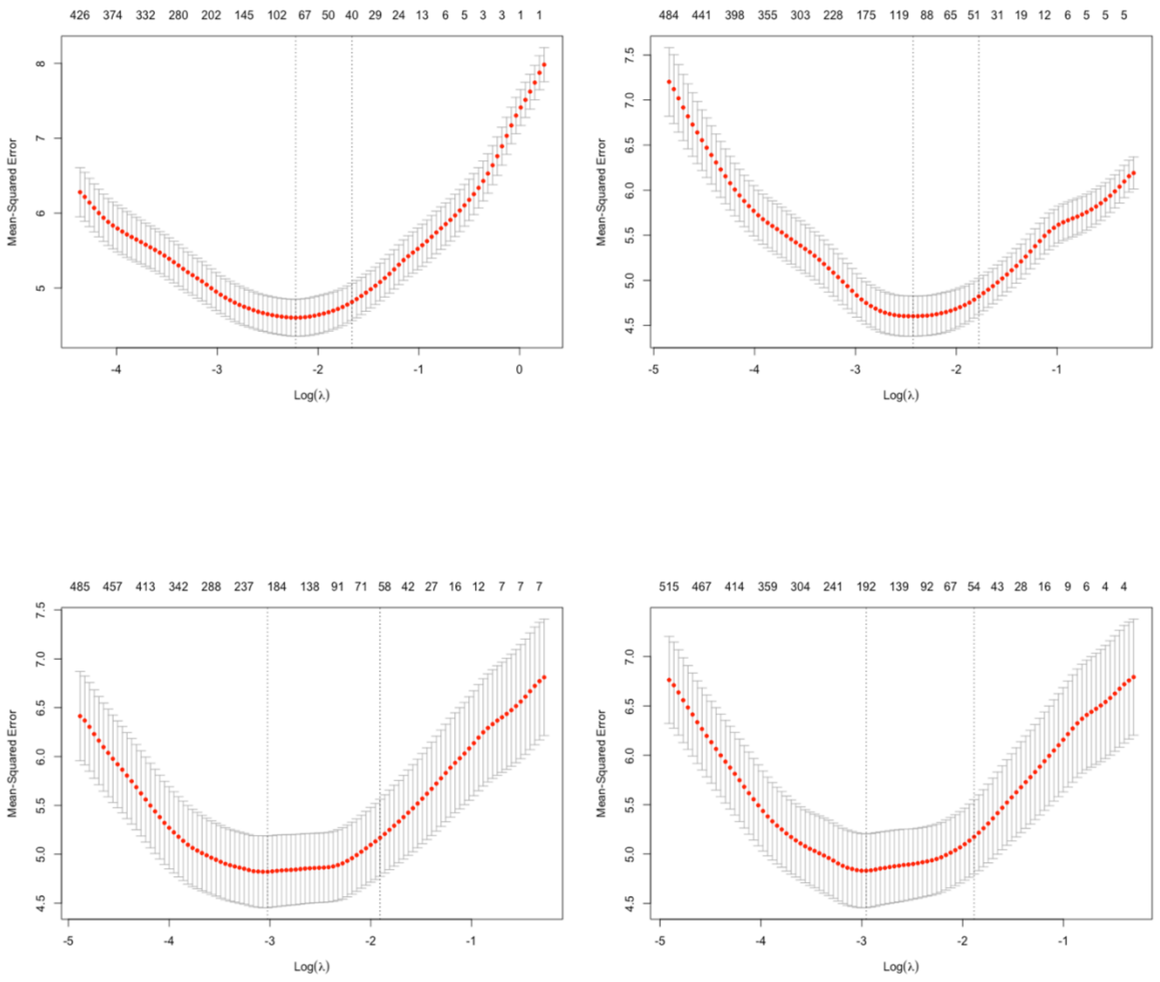

ESM Figure 2. The cross-validation curve plot with upper and lower standard deviation curves along the λ sequence (error bars) in the RISC cohort using M as a binary variable considering the lower quartile of M-value compared to the remaining 3/4 of the cohort. Model 1 performed only with proteins; Model 2 performed with proteins and age, sex, center, and body mass index; Model 3 performed with proteins and age, sex, center, body mass index, lipids, and systolic blood pressure; Model 4 performed with proteins and common clinical covariates: body mass index, lipids, and systolic blood pressure.

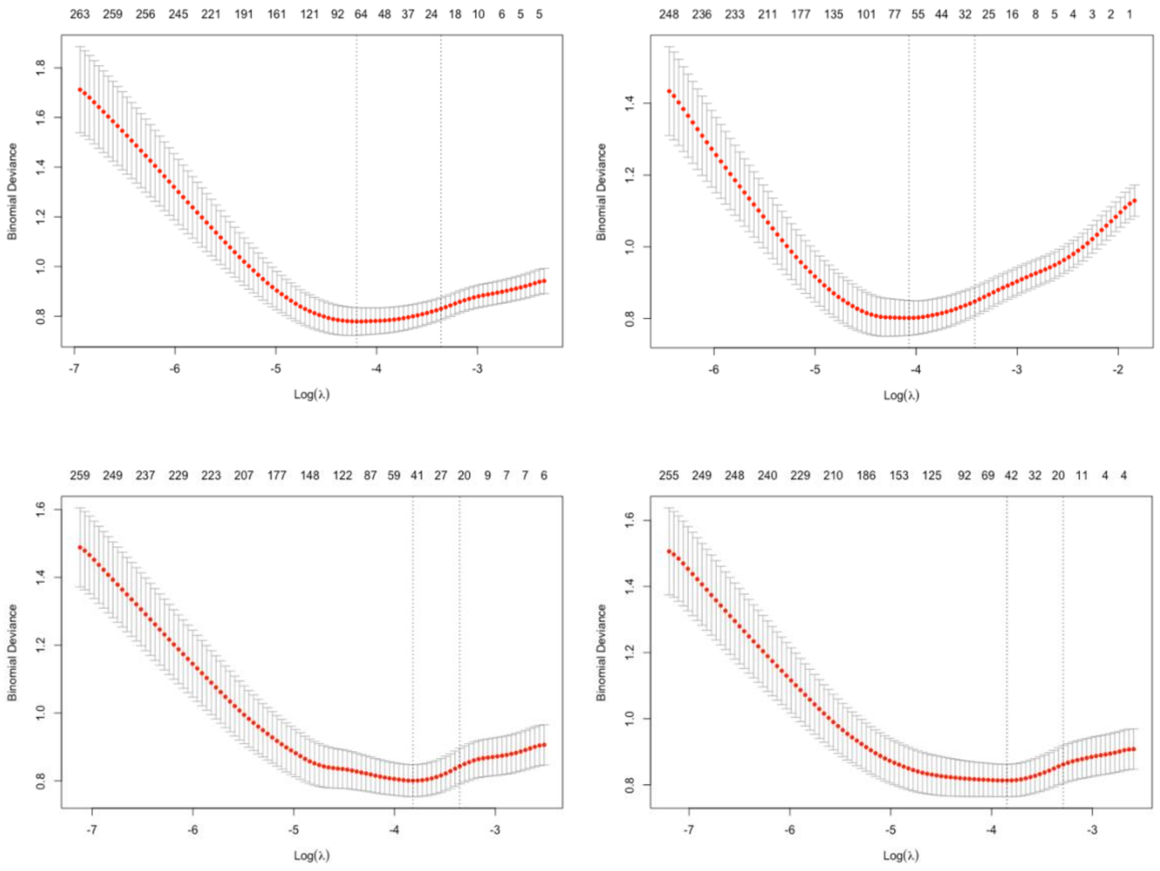

ESM Figure 3. The cross-validation curve plot with upper and lower standard deviation curves along the λ sequence (error bars) in the ULSAM cohort using M as a continuous variable. Model 1 performed only with proteins; Model 2 performed with proteins and age, sex, center, and body mass index; Model 3 performed with proteins and age, sex, center, body mass index, lipids, and systolic blood pressure; Model 4 performed with proteins and common clinical covariates: body mass index, lipids, and systolic blood pressure.

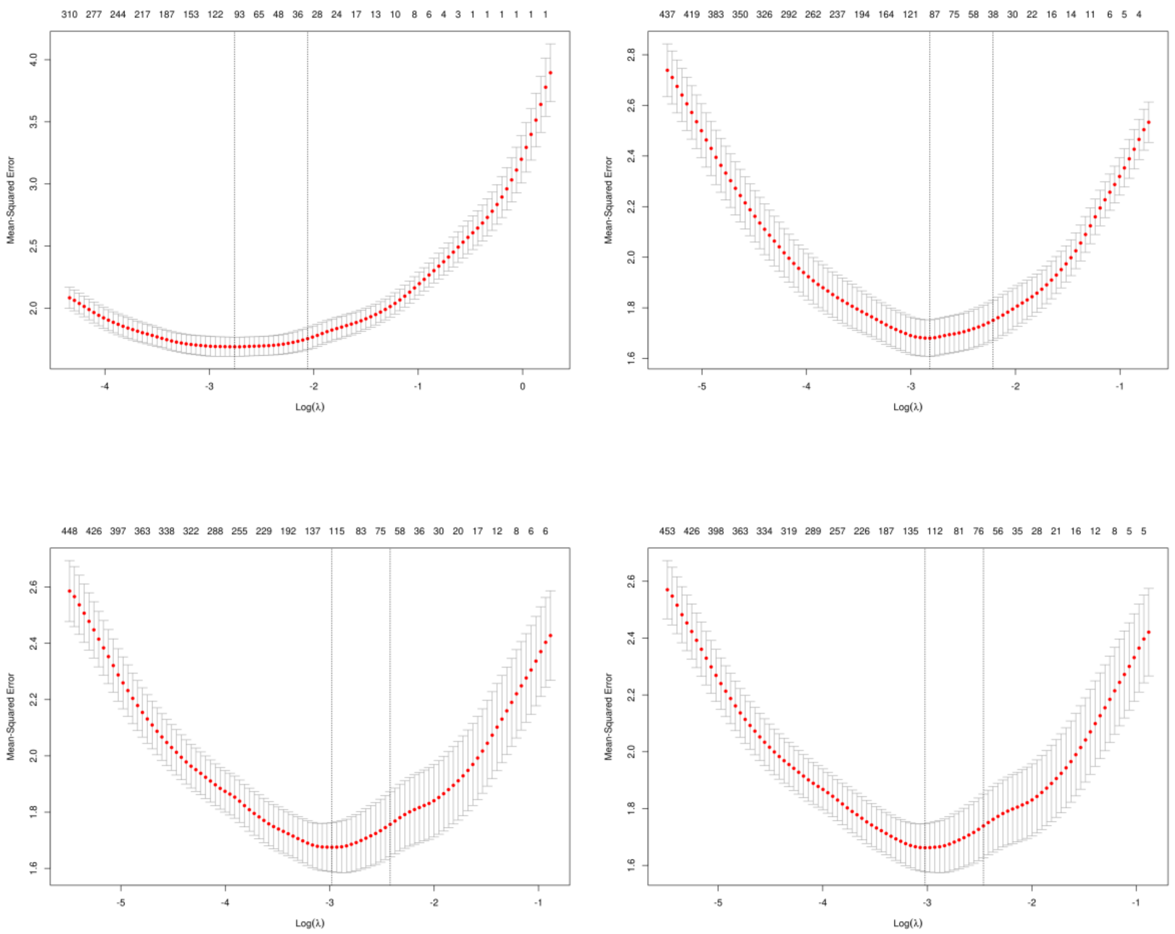

ESM Figure 4. The cross-validation curve plot with upper and lower standard deviation curves along the λ sequence (error bars) in the ULSAM cohort using M as a binary variable considering the lower quartile of M-value compared to the remaining 3/4 of the cohort. Model 1 performed only with proteins; Model 2 performed with proteins and age, sex, center, and body mass index; Model 3 performed with proteins and age, sex, center, body mass index, lipids, and systolic blood pressure; Model 4 performed with proteins and common clinical covariates: body mass index, lipids, and systolic blood pressure.

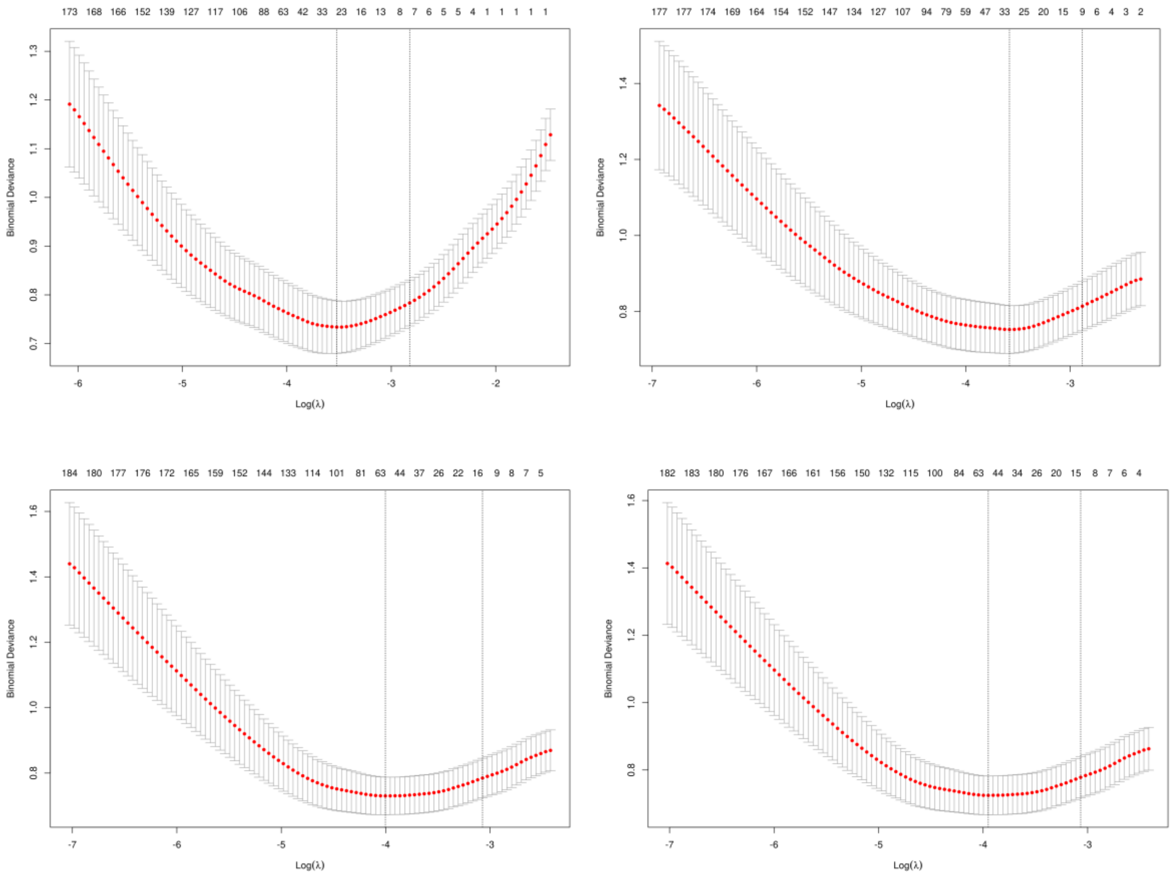

ESM Figure 5 a-d. Root Mean Square Error (RMSE) using M as a continuous variable and Mean Square Error (MSE) using M as a binary variable in all the models performed using the training and the test datasets from the same cohort: RISC (a) and ULSAM (b) and using the training in one cohort and testing dataset in the other cohort: RISC versus ULSAM (c) and ULSAM versus RISC (d).

a)

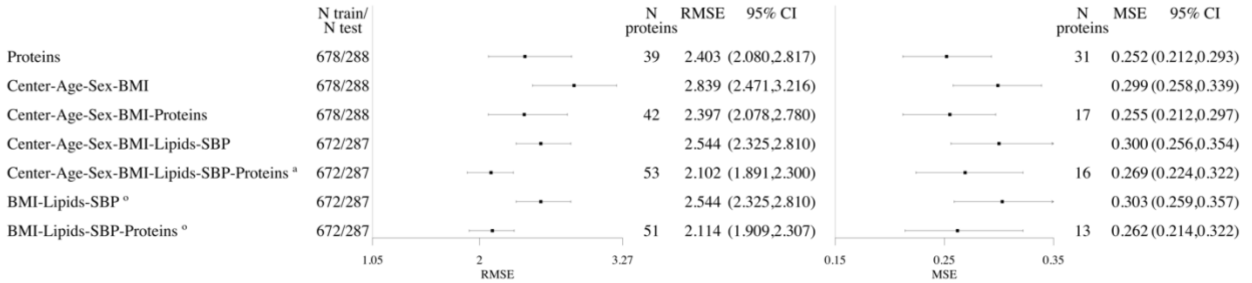

b)

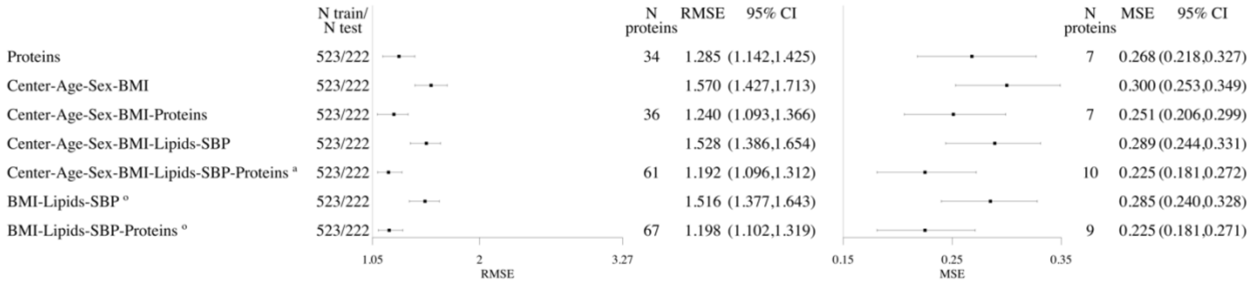

c)

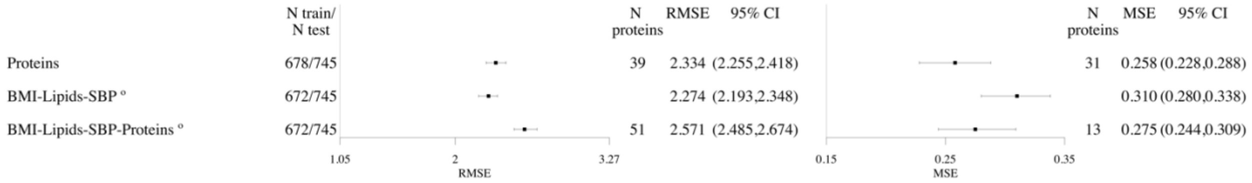

d)

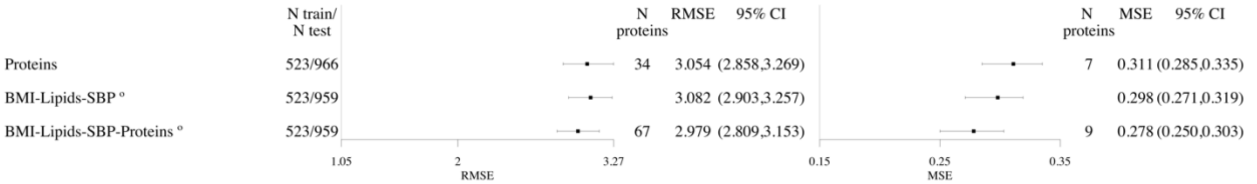

In ULSAM age is a limited covariate (69-73 years old) and center and sex were fixed.

[a] A priori main model. [o] Models with the common covariates to both cohorts. BMI was selected in all the only-covariates models performed. Lipids were additionally selected in the only-covariates models in ULSAM only when M was used as a continuous variable.

Members of the RISC investigators study group

Prof Ele Ferrannini - Department of Internal Medicine, University of Pisa, Pisa, Italy

Dr Michaela Kozakova - Department of Clinical and Experimental Medicine, University of Pisa, 56124 Pisa, Italy

Dr Amalia Gastaldelli - Institute of Clinical Physiology, National Research Council-CNR, Pisa 56100, Italy

Dr Simon Coppack -Academic Medical Unit, The Royal London Hospital, Whitechapel, London, UK

Dr Beverley Balkau - Inserm U1018, Institut Gustave Roussy, Center for Research in Epidemiology and Population Health, Villejuif, France

Dr Jacqueline Dekker - EMGO Institute, Vrije Universiteit Amsterdam Amsterdam, The Netherlands

Prof Mark Walker - Institute of Cellular Medicine, William Leech Building, Medical School, Newcastle University, Newcastle, NE2 4HH, UK

Dr Andrea Mari - Institute of Neuroscience, National Research Council, Padova, Italy

Dr Andrea Tura - Metabolic Unit, CNR Institute of Neuroscience, Padua, Italy

Prof Martine Laville - Department of Endocrinology, Claude Bernard University, Lyon, France.

Prof Henning Beck -Nielsen Steno Diabetes Center Odense, Odense University Hospital, Odense, Denmark

Prof John Nolan - Professor (adjunct), Endocrinology & Metabolism, Trinity College Dublin, Ireland

Prof Geremia Bolli - Department of Medicine and Surgery, Section of Endocrinology and Metabolism, University of Perugia, Perugia, Italy

Prof Alain Golay - Service d'enseignement thérapeutique pour maladies chroniques, HUG, Genève, Switzerland

Dr Thomas Konrad - Stoffwechselzentrum Rhein-Main Eschersheimer Landstr. 10, 60322 Frankfurt am Main, Germany

Dr Peter Nilsson and Dr Olle Melander - Department of Clinical Sciences, Lund University, Skåne University Hospital, S-20502 Malmö, Skåne, Sweden

Prof Geltrude Mingrone - King's College London, Department of Diabetes, School of Life Course Science, London, UK
Dr Colin Perry - Institute of Cardiovascular and Medical Sciences, British Heart Foundation, Glasgow Cardiovascular Research Center, University of Glasgow, United Kingdom
Prof John Petrie - School of Health and Wellbeing, College of Medical, Veterinary and Life Sciences, University of Glasgow, Glasgow, UK
Dr Michael Krebs - Abteilung für Endokrinologie und Stoffwechsel, Med .Univ., Wien, Austria.
Dr Rafael Gabriel - Foundation World Community for Prevention of Diabetes (WCPD), Madrid, Spain
Dr Asimina Mitrakou - 2nd Dept. of Internal Medicine, Propaedeutic, University of Athens, Greece
Dr Piermarco Piatti - Unità Funzionale di Cardio-Metabolismo e Trials Clinici dell’Unità Operativa di Medicina Generale a indirizzo Diabetologico ed Endocrino Metabolico dell’IRCCS Ospedale San Raffaele, Milano, Italy
Prof Nebojsa Lalic - Clinic for Endocrinology, Diabetes and Metabolic Diseases, University Clinical Center of Serbia, Faculty of Medicine, University of Belgrade, Belgrade, Serbia
Prof Marku Laakso - Institute of Clinical Medicine, Internal Medicine, University of Eastern Finland, Kuopio, Finland
